# Morphological and phylogenetic analyses reveal three new species of Chaetosphaeriaceae from China

**DOI:** 10.3897/mycokeys.131.187694

**Published:** 2026-04-16

**Authors:** Sen Chen, Haimei Zhao, Wen Zheng, Long Han

**Affiliations:** 1 Guizhou Key Laboratory of Microbio and Infectious Disease Prevention & Control, Guizhou Medical University, Guiyang 561113, Guizhou, China Engineering Research Center for Bio-Perception Materials of Guizhou Province, Guizhou Medical University Guiyang China; 2 Engineering Research Center for Bio-Perception Materials of Guizhou Province, Guizhou Medical University, Guiyang 561113, Guizhou, China Guizhou Key Laboratory of Microbio and Infectious Disease Prevention & Control, Guizhou Medical University Guiyang China; 3 State Key Laboratory of Discovery and Utilization of Functional Components in Traditional Chinese Medicine & school of pharmaceutical science, Guizhou Medical University, Guiyang 561113, Guizhou, China State Key Laboratory of Discovery and Utilization of Functional Components in Traditional Chinese Medicine & school of pharmaceutical science, Guizhou Medical University Guiyang China; 4 The Key Laboratory of Optimal Utilization of Natural Medicine Resources (The Union Key Laboratory of Guiyang City-Guizhou Medical University), Guizhou Medical University, Guiyang 561113, Guizhou, China The Key Laboratory of Optimal Utilization of Natural Medicine Resources (The Union Key Laboratory of Guiyang City-Guizhou Medical University), Guizhou Medical University Guiyang China; 5 Engineering Research Center of Microbiology and Biochemical Pharmaceutical, Guizhou Medical University, Guiyang 561113, Guizhou, China Engineering Research Center of Microbiology and Biochemical Pharmaceutical, Guizhou Medical University Guiyang China

**Keywords:** 3 new taxa, asexual morph, Chaetosphaeriales, sporidesmium-like taxa, taxonomy

## Abstract

During an ongoing taxonomic investigation of microfungi in southern China, three novel taxa belonging to the family Chaetosphaeriaceae are introduced based on morphological characteristics and phylogenetic analyses of combined ITS, LSU and *tef*1-α sequence data. Two of the new species, *Brunneodinemasporium septatum* and *Codinaea yulinensis*, possess phialidic asexual morphs, whereas the third new species, *Pseudolomaantha yulinensis*, is characterized by a non-phialidic, sporidesmium-like asexual morph. Detailed morphological descriptions and illustrations are provided for all taxa, together with notes comparing them to morphologically similar species. Phylogenetic analyses support their placement within Chaetosphaeriaceae and confirm their status as distinct species.

## Introduction

Chaetosphaeriaceae was validly established by Réblová et al. (1999) to accommodate *Chaetosphaeria* and its relatives, such as *Ascocodinaea*, *Melanochaeta*, and *Melanopsammella*. Since then, the family has expanded considerably and now comprises more than 100 accepted genera, making it one of the largest families in Sordariomycetes (Lin et al. 2019; Yang et al. 2019; Réblová et al. 2020, 2021a, 2021b, 2022, 2024; Wu and Diao 2022; Zhang et al. 2022; Réblová and Nekvindová 2023; Yeh et al. 2024). Members of Chaetosphaeriaceae are widely distributed in both terrestrial and freshwater habitats, where they occur primarily as saprobes on decaying leaves, fruits, branches, bark, and wood (Hughes and Kendrick 1968; Réblová et al. 1999, 2024; Lin et al. 2019; Wu and Diao 2022).

*Brunneodinemasporium* was introduced by Crous et al. (2012) based on the type species *B. brasiliense*, which is characterized by dinemasporium-like asexual morph. Currently, three species are accepted in this genus, namely *B. brasiliense*, *B. jonesii*, and *B. sinense*, based on morphological and phylogenetic evidence (Crous et al. 2012; Lu et al. 2016; Wu and Diao 2022). All species are saprobic on dead plant material and have been reported from Brazil and China (Crous et al. 2012; Lu et al. 2016; Wu and Diao 2022).

*Codinaea* was established by Maire (1937) with *C. aristata* as the type species. Although the taxonomy of the genus remained confused for a long time, it has undergone multiple revisions and is now well clarified (Hughes and Kendrick 1968; Réblová 2004; Barbosa et al. 2016; Réblová et al. 2020, 2021b; Wu and Diao 2022). *Codinaea*, a heterogeneous and hyphomycetous genus, is characterized by dematiaceous conidiophores, phialidic conidiogenous cells and falcate to lunate or oblong-falcate, hyaline, setulate conidia (Réblová et al. 2020, 2021b; Wu and Diao 2022; Zhang et al. 2022). Species of *Codinaea* are widely distributed and primarily saprobic, occurring on decaying leaves, fruits, branches, bark, and wood (Hughes and Kendrick 1968; Réblová et al. 2020, 2021b; Wu and Diao 2022).

*Pseudolomaantha* is a monotypic genus introduced by Zhang et al. (2025b) to accommodate *P. thailandica*, which was isolated from dead bamboo stems in Thailand, based on morphological characteristics and phylogenetic analyses. The genus is characterized by a sporidesmium-like morph with macronematous, mononematous conidiophores, monoblastic conidiogenous cells, and pyriform to obclavate, rostrate conidia with apical appendage (Zhang et al. 2025b).

In this study, three asexual taxa associated with dead plant substrates were obtained from Guangxi Province in China, namely *Brunneodinemasporium septatum*, *Codinaea yulinensis* and *Pseudolomaantha yulinensis*. The recognition of these new taxa is based on morphological comparisons and multi-gene phylogenetic analyses.

## Material and methods

### Collections, isolation and conservation

Samples of dead plant tissues were randomly collected from Guangxi Province, China. Specimens were packed in plastic bags for transport to the laboratory, and the collection data (e.g., date and habitat), were recorded. Morphological observations of the fungal colonies and fruiting bodies were conducted using a stereomicroscope (SteREO Discovery, V12, Carl Zeiss Microscopy GmBH, Germany; VHX-7000, Keyence, Japan). Fungal structures were examined and photographed using a Nikon Eclipse Ni compound microscope (Nikon, Japan) and photographed with a Nikon DS-Ri2 digital camera (Nikon, Japan) attached to the microscope. Tarosoft^®^ Image Frame Work Version 0.9.7 was used for the measurement of the photomicrograph. Photo-plates were made with Adobe Photoshop CC 2019 v. 19.1.6 (Adobe Systems, USA).

Single-spore isolation was carried out onto water agar (WA; 16 g/L distilled water), and germinated spores were transferred to potato dextrose agar (PDA; 39 g/L distilled water, Difco potato dextrose) to obtain pure cultures following the method in (Chomnunti et al. 2014). Dried specimens were deposited in the Herbarium of Guizhou Academy of Agricultural Sciences (GZAAS), Guiyang, China. Pure cultures were deposited in the Guizhou Culture Collection (GZCC), Guiyang, China. Index Fungorum identifiers were obtained following the guidelines in Index Fungorum (http://www.indexfungorum.org/Names/Names.asp; accessed on 31 January 2026).

### DNA extraction, PCR amplification and sequencing

Fresh fungal mycelia (200–500 mg) were scraped from grown on PDA plates and transferred to a microcentrifuge tube using sterilized needles for genomic DNA extraction. Genomic DNA was extracted using the Biospin Fungus Genomic DNA Extraction Kit (Biospin Fungus Genomic DNA Extraction Kit, BioFlux®, Shanghai, China). Three gene regions, the internal transcribed spacer (ITS), the large subunit of ribosomal DNA (LSU), and the translation elongation factor 1 (*tef*1-α), were amplified using the primer pairs ITS5 and ITS4 (White et al. 1990), LR0R and LR5 (Vilgalys and Hester 1990), and EF1-983F and EF1-2218R (Rehner and Buckley 2005), respectively. Polymerase chain reaction (PCR) was performed in a 50 µL reaction mixture containing 2 µL of DNA template, 2 µL of each forward and reverse primer (10 µM), 25 µL of 2× Taq PCR Master Mix with blue dye (Sangon Biotech, China), and 19 µL of distilled–deionized water. Amplification conditions for the ITS and LSU regions followed the protocol described by Zhang et al. (2022). The following thermo-cycling parameters were used for *tef*1-α: initial denaturation at 94 °C for 3 min, followed by 40 cycles of denaturation at 94 °C for 45 s, annealing at 53 °C for 50 s, elongation at 72 °C for 1 min and a final extension period for 10 min at 72 °C. Purification and sequencing of PCR products were performed by Beijing Qingke Biotechnology Co., Ltd.

### Phylogenetic analyses

Raw sequence chromatograms were checked by BioEdit v. 7.1.3.0 (Hall 1999). SeqMan v. 7.0.0 (DNASTAR, Madison, WI, USA) were used to assemble forward and reverse sequences. BLASTn searches in NCBI GenBank (https://blast.ncbi.nlm.nih.gov/Blast.cgi) were used to determine closely related strains to our new taxa. Consequently, representative sequence data from Chaetosphaeriaceae were obtained from GenBank, referring to previous studies (Réblová et al. 2021b; Zhang et al. 2022, 2025b; Wu and Diao 2022). The sequences were aligned using the online multiple alignment program MAFFT v.7 (http://mafft.cbrc.jp/alignment/server/, accessed January 2026; Katoh et al. 2019). Trimal v1.2 (Capella-Gutiérrez et al. 2009) was used to remove ambiguously aligned regions and uninformative positions with the “gt = 0.6” option. ITS, LSU and *tef*1-α sequences were combined using SequenceMatrix 1.7.8 (Vaidya et al. 2011). Sequences derived in this study were deposited in GenBank (Table 1).

**Table 1.** Taxa used in this study with the corresponding GenBank accession numbers.

Taxon	Strain number	Status	GenBank Accessions		
			ITS	LSU	*tef*1-α
*Brachydictyochaeta antillana*	NN058987		OL627951	OL655147	–
*Brachydictyochaeta bulliformis*	NN076027		OL628023	OL655155	–
*Brunneodinemasporium brasiliense*	CBS 112007	T	JQ889272	JQ889288	–
*Brunneodinemasporium jonesii*	GZCC 16-0050	T	KY026058	KY026055	–
*Brunneodinemasporium jonesii*	ZHKUCC 24-1231		PX663211	PX667655	–
*Brunneodinemasporium septatum*	GZCC 23-0768	T	PX971465	PX971469	PX979698
*Brunneodinemasporium sinense*	CGMCC 3.20659	T	OL627952	OL655148	–
*Cacumisporium acutatum*	CBS 101315	T	OR134682	OR134626	OR130762
*Cacumisporium capitulatum*	CBS 101313		OR134683	OR134627	OR130763
*Caliciastrum bicolor*	ICMP 15136	T	OR134689	OR134633	OR130769
*Caligospora dilabens*	CBS 734.83	T	OR134691	OR134636	OR130771
*Caligospora pannosa*	CBS 551.89	T	OR134692	OR134637	OR130772
*Catenularia catenulata*	DLUCC 0891	T	MK828637	MK835838	MN194088
*Catenularia minor*	PRM 900544	T	MW987827	MW987822	OL653993
*Chaetosphaeria innumera*	CBS 145639		OP455358	OP455464	OP465036
*Chaetosphaeria mangrovei*	MCD 069	T	MG813821	MG813820	–
*Chloridium bellum*	CBS 709.73A	T	OP455360	OP455466	OP464934
*Chloridium gamsii*	CBS 667.75	T	OP455415	OP455522	OP464990
*Codinaea acaciae*	KUNCC 23-13363		OR159317	OR159335	OR194109
*Codinaea amazonensis*	MUCL 41171		OL654076	OL654133	OL653996
*Codinaea aseptata*	GZCC 22-0081	T	ON502897	ON502890	–
*Codinaea assamica*	CBS 139907	T	OL654077	OL654134	OL653997
*Codinaea clavatophora*	NN47943	T	OL627680	–	–
*Codinaea dinghushanensis*	NN54218	T	OL627723	–	–
*Codinaea dwaya*	CBS 261.77	T	OL654078	OL654135	OL653998
*Codinaea ellipsoidea*	MFLUCC 18-1574	T	MK828628	MK835828	MN194080
*Codinaea fanglanii*	NN58983	T	OL627950	OL655146	–
*Codinaea fecunda*	BCRC FU31889	T	–	–	PP471528
*Codinaea fertilis*	IMI 233824		OL654080	OL654137	OL654000
*Codinaea gonytrichodes*	CBS 593.93		AF178556	AF178556	OL654001
*Codinaea kendrickii*	NN057551	T	OL627885	–	–
*Codinaea latispora*	NN077341	T	OL628329	–	–
*Codinaea lignicola*	DLUCC 0899	T	MK828630	MK835830	MN194081
*Codinaea lithocarpi*	MFLUCC 17-2228	T	NR_171095	NG_073858	–
*Codinaea oxenbolliae*	NN077595	T	OL628418	–	–
*Codinaea pandanicola*	KUMCC 16-0153	T	MH388338	MH376710	MH388373
*Codinaea paniculata*	CBS 145098	T	MT118230	MT118201	OL654002
*Codinaea phasma*	CBS 147516	T	OL654081	OL654138	OL654006
*Codinaea pyriformis*	NN045929	T	OL627653	OL655056	–
*Codinaea siamensis*	MFLUCC 15-0614	T	KX609955	KX609952	OP473066
*Codinaea simaoensis*	NN076046	T	OL628033	–	–
*Codinaea terminalis*	GZCC 18-0085	T	MN104613	MN104624	–
*Codinaea trisetula*	NN044720	T	OL627631	OL655039	–
*Codinaea yulinensis*	GZCC 23-0769	T	PX971466	PX971470	PX979699
*Codinaea vermispora*	YMF1.4260	T	MK165444	MK165442	–
*Codinaeella lambertiae*	CBS 143419	T	OL654084	OL654141	OL654009
*Codinaeella minuta*	CBS 280.59		OL654090	OL654147	OL654016
*Codinaeella parvilobata*	CBS 144536	T	OL654100	OL654157	OL654027
*Craspedodidymum elatum*	NN042874		OL627547	OL655004	–
*Curvichaeta curvispora*	ICMP 15115	T	OR134705	OR134650	OR130785
*Dictyochaeta callimorpha*	ICMP 15130		MT454483	MT454498	MT454673
*Dictyochaeta fuegiana*	ICMP 15153	T	MT454487	EF063574	MT454677
*Menisporopsis pirozynskii*	MUCL 47217		MW984579	MW984561	OL654047
*Menisporopsis theobromae*	MUCL 41079		MW984580	MW984562	OL654048
*Multiguttulispora dimorpha*	CBS 140002		MW984582	MW984564	OL654049
*Multiguttulispora triseptata*	IMI 353690		MW984584	MW984566	OL654050
*Neopseudolachnella acutispora*	MAFF 244358	T	AB934065	AB934041	AB934091
*Neopseudolachnella magnispora*	MAFF 244359	T	AB934066	AB934042	AB934092
*Paliphora intermedia*	CBS 896.97	T	MH862682	EF204501	–
*Papillospora hebetiseta*	CBS 102340	T	AF178549	AF178549	OL653994
*Paragaeumannomyces panamensis*	S.M.H. 3596	T	AY906948	MT118218	–
*Paragaeumannomyces rubicundus*	S.M.H. 3221	T	MT118242	MT118224	–
*Phialoturbella calva*	ICMP 23826	T	MW984585	MW984567	OL654052
*Phialoturbella lunata*	MFLUCC 18-0642	T	MK828624	MK835824	MN194077
*Pseudolachnea fraxini*	CBS 113701	T	JQ889287	JQ889301	AB934096
*Pseudolachnea hispidula*	MAFF 244365		AB934072	AB934048	AB934098
*Pseudolachnella asymmetrica*	MAFF 244366	T	AB934073	AB934049	AB934099
*Pseudolachnella scolecospora*	MAFF 244379		AB934086	AB934062	AB934112
*Pseudolomaantha thailandica*	MFLUCC 24-0521	T	PQ625465	PQ625467	–
*Pseudolomaantha yulinensis*	GZCC 23-0770	T	PX971467	PX971471	PX979700
*Pseudolomaantha yulinensis*	GZCC 23-0771	T	PX971468	PX971472	PX979701
*Tracylla aristata*	CBS 141404	T	OL654129	OL654186	OL654068
*Tracylla eucalypti*	CBS 144429	T	OL654130	OL654187	OL654069

Note: status: T denotes type strains; “–” indicates no data are available in GenBank.

Maximum likelihood (ML) analysis was conducted using RAxML-HPC v. 8.2.12 tool via the CIPRES science Gateway CIPRES science Gateway V. 3.3 (https://www.phylo.org/portal2/home.action; Miller et al. 2010). One thousand non-parametric bootstrap iterations were run, and the best-scoring tree was selected from suboptimal trees under the GTRGAMMA substitution model.

Bayesian inference (BI) analysis was performed in MrBayes 3.2.7a (Ronquist et al. 2012) via the CIPRES (Miller et al. 2010) web portal. The best-fit substitution model GTR + I + G was decided for each locus by MrModeltest 2.3 (Nylander 2004) under the Akaike Information Criterion (AIC). Bayesian posterior probabilities (PPs) (Rannala and Yang 1996; Huelsenbeck and Ronquist 2001) were evaluated based on Markov Chain Monte Carlo (MCMC) sampling (Rannala and Yang 1996; Huelsenbeck and Ronquist 2001). Two parallel runs of four simultaneous Markov chains were performed for 1 million generations, with trees sampled every 100^th^ generation, resulting in 10,000 trees.

Phylogenetic trees were visualized by FigTree v.1.4.4 (Rambaut 2014), and the layouts were reorganized online (https://www.chiplot.online/) using the methods described by Xie et al. (2023) and finalized with Adobe Illustrator CS6 software (Adobe Systems, USA).

### Phylogenetic analysis results

**Figure 1.**
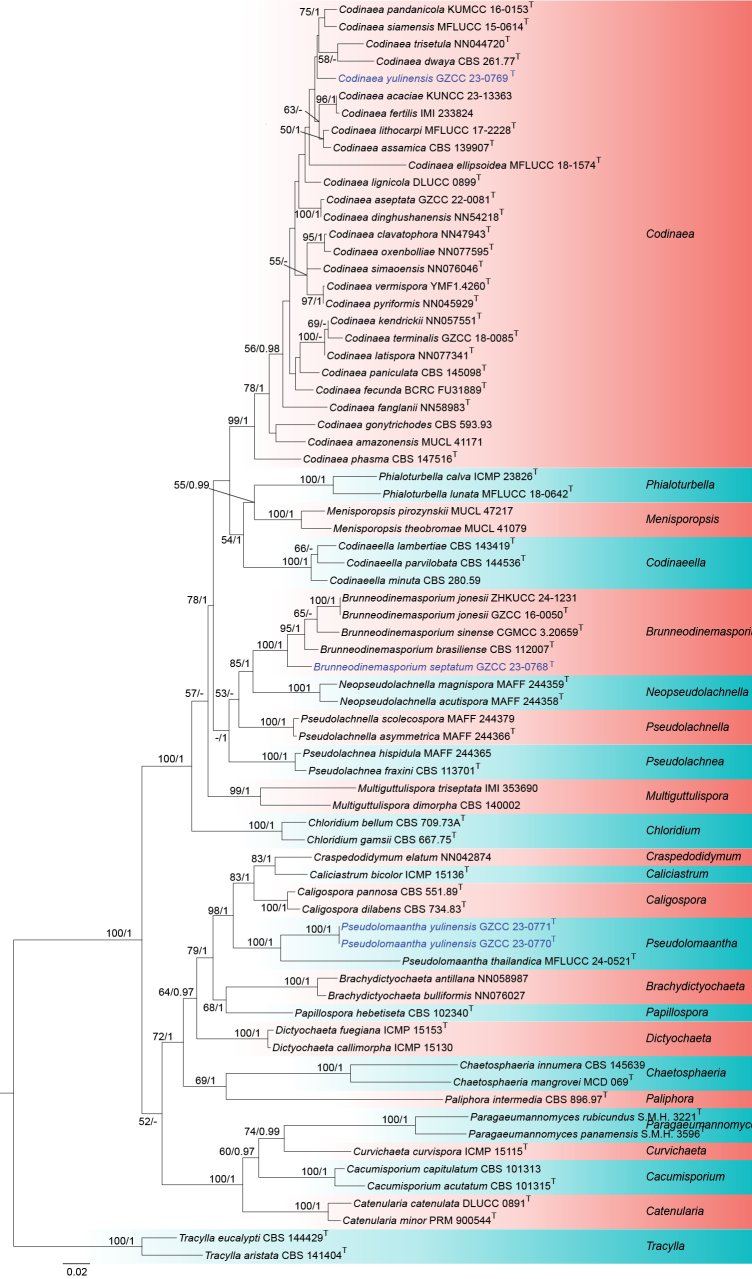
Phylogram generated from maximum likelihood analysis based on combined ITS-LSU-*tef*1-α sequence data. Bootstrap support values for ML equal to or greater than 50% and Bayesian posterior probabilities (PPs) equal to or greater than 0.95 were indicated above or below the nodes as ML/PP. *Tracylla aristata* (CBS 141404) and *T. eucalypti* (CBS 144429) were selected as the outgroup taxa. Type strains are marked with T after the strain number. The newly obtained sequences are indicated in blue.

Phylogenetic relationships of three Chaetosphaeriaceae species were assessed based on combined ITS-LSU-*tef*1-α sequence data. The combined analyses included 73 strains, with *Tracylla aristata* (CBS 141404) and *T. eucalypti* (CBS 144429) serving as the outgroup taxa. The final alignment comprised 2,770 characters, including gaps, and consisted of combined ITS (1–484 bp), LSU (485–1837 bp), and *tef*1-α (1838–2770 bp) sequence data. The dataset included 1,056 distinct alignment patterns, with 24.61% comprising undetermined characters or gaps. The best-scoring RAxML tree is shown in Fig. 1, with a final likelihood value of -23750.909940. The analyzed ML and Bayesian trees were similar in topology and did not conflict significantly.

The multi-gene analyses (Fig. 1) showed that our four newly generated isolates nested within Chaetosphaeriaceae, and represented three independent lineages corresponding to three novel species in *Brunneodinemasporium*, *Codinaea*, and *Pseudolomaantha*, respectively. *Brunneodinemasporium septatum* formed a distinct clade basal to the other three *Brunneodinemasporium* species, with 100% ML and 1 PP values. *Codinaea yulinensis* sp. nov. clustered with other *Codinaea* species and is closely related to *C. dwaya*, *C. pandanicola*, *C. siamensis*, and *C. trisetula*. *Pseudolomaantha yulinensis* sp. nov. is sister to *P. thailandica*, also supported by 100% ML and 1 PP.

## Taxonomy

### Brunneodinemasporium septatum

Taxon classificationFungiChaetothyrialesChaetothyriaceae

S. Chen, W. Zheng & L. Han sp. nov.

1313871F-DE7D-5055-BDE2-742032C7EC82

Index Fungorum: IF905052

Fig. 2

#### Etymology.

The epithet “septatum” refers to the septate conidia.

**Figure 2.**
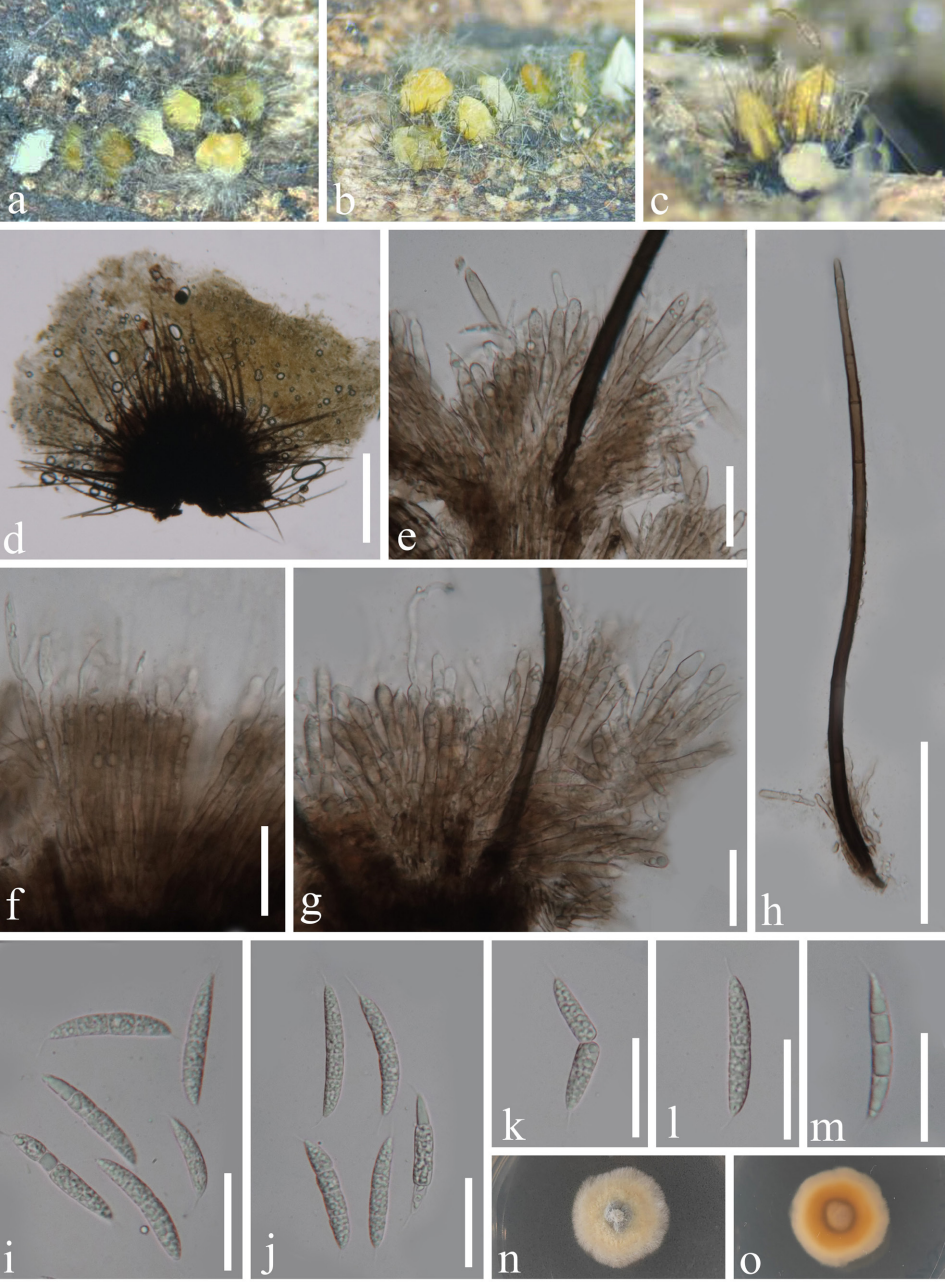
*Brunneodinemasporium septatum* (GZAAS 23-0849, holotype). **a–d**. Acervular conidiomata with setae; **e–g**. Conidiophores with conidiogenous cells; **h**. Setae; **i–m**. Conidia; **n, o**. Pure culture from above and below. Scale bars: 300 µm (**d**); 30 µm (**e–g, i–m**); 100 µm (**h**).

#### Holotype.

GZAAS 23-0849.

#### Description.

***Saprobic*** on dead wood in a terrestrial habitat. **Asexual morph**: ***Colonies*** on natural substrate superficial, effuse, sporodochial, mostly in groups or sometimes scattered, pulvinate, cupulate, dark brown to black, with a white to yellow slimy conidial mass in the center, setose. ***Mycelium*** is composed of mostly immersed, brown, septate, hyphae. ***Setae*** sterile, abundant, septate, straight or flexuous, subulate, acutely pointed, unbranched, brown to black, becoming paler towards the apex, smooth-walled, up to 530 μm long, 5.7–8.3 μm wide, arising from basal stroma. ***Conidiophores*** macronematous, formed a tightly packed palisade covering the surface of the stroma, septate, straight or flexuous, cylindrical, unbranched, brown, up to 130 μm long. ***Conidiogenous cells*** integrated, determinate, monophialidic, smooth-walled, subcylindrical, subhyaline to pale brown, 9.5–29.5 µm long × 4.5–6.5 µm wide (x̄ = 19.2 × 5.5 μm, n = 20). ***Conidia*** acrogenous, 1–3-septate, straight or curved, fusiform, guttulate, hyaline to subhyaline, 34–43(–46.3) µm long × 5.2–7 µm wide (x̄ = 39 × 6.3 μm, n = 30), with unbranched, tubular, filiform, hyaline, 5.5–6.3 µm long setula at each end. **Sexual morph**: not observed.

#### Culture characteristics.

Conidia germinated on WA within 24 hours at 25 °C. Germ tubes were produced from the base and the upper. Colonies growing on PDA, reaching 25–30 mm diameter in 25 days, circular, with entire margin, flat, with a protuberance in the center, fuzzy, creamy-white in above, in reverse yellowish in the center, yellowish-brown to yellowish from the middle ring to margin.

#### Material examined.

China • Guangxi Province, Yulin City, Minle Town, on a dead wood in a forest near the roadside, 16 July 2023, S. Chen, 55.2 (GZAAS 23-0849, **holotype)**, ex-type GZCC 23-0768.

#### Additional sequence.

SSU: PX971510.

#### Notes.

Phylogenetic analysis based on multiple genes (Fig. 1) showed that *B. septatum* represents a distinct lineage within *Brunneodinemasporium*, forming a basal clade to *B. brasiliense*, *B. jonesii* and *B. sinense*, with maximal support (100% ML and 1 PP). Morphologically, *B. septatum* is similar to these three *Brunneodinemasporium* species in the shapes of conidiomata, conidiophores, conidiogenous cells and conidia (Crous et al. 2012; Lu et al. 2016; Wu and Diao 2022). However, it differs from *B. brasiliense*, *B. jonesii* and *B. sinense* in producing septate conidia that are larger than those of these species (34–43 µm × 5.2–7 µm *vs*. 18–19 µm × 2.5–3 µm *vs*. 6–9.5 µm × 1.5–2 µm *vs*. 20–23 × 2–2.5 µm, respectively).

### Codinaea yulinensis

Taxon classificationFungiChaetosphaerialesChaetosphaeriaceae

S. Chen, W. Zheng & L. Han sp. nov.

17A629BC-408A-51E9-9994-714E63BB3E22

Index Fungorum: IF905053

Fig. 3

#### Etymology.

The epithet “*yulinensis*” refers to the location (Yulin city) from which the fungus was collected.

**Figure 3.**
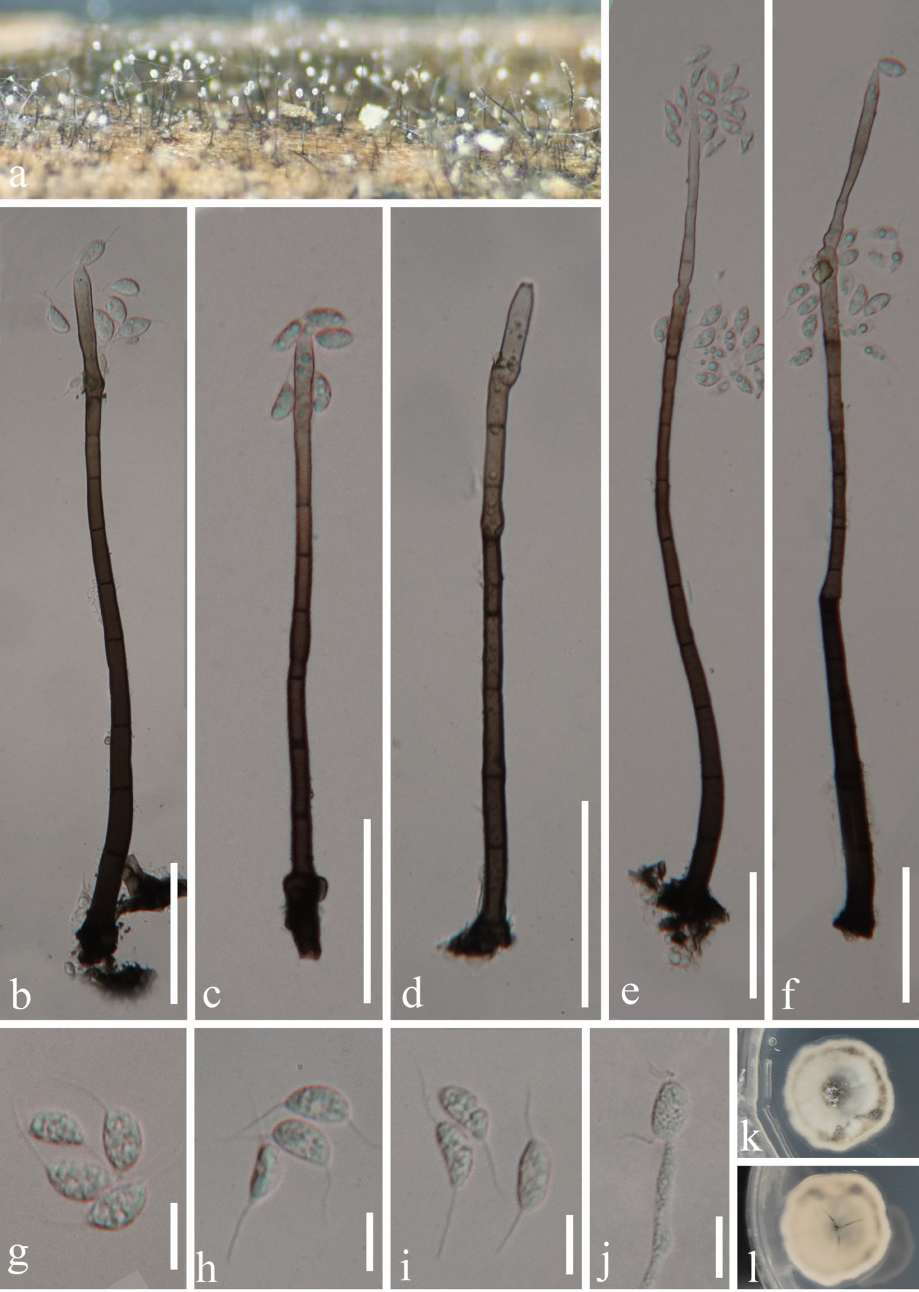
*Codinaea yulinensis* (GZAAS 23-0850, holotype). **a**. Colonies on the host substrate; **b–f**. Conidiophores with conidiogenous cells (arrows showing conidiogenous loci); **g–i**. Conidia; **j**. Germinating conidium; **k, l**. Pure culture from above and below. Scale bars: 50 µm (**b–f**); 10 µm (**g–j**).

#### Holotype.

GZAAS 23-0850.

#### Description.

Saprobic on dead rachis of an unidentified fern in a terrestrial habitat. **Asexual morph**: ***Colonies*** on natural substrate superficial, effuse, scattered, brown, with white conidial masses on the apex of conidiophores. ***Mycelium*** is composed of mostly immersed, brown, septate hyphae. ***Conidiophores*** macronematous, mononematous, with intercalary conidiogenous loci, solitary, septate, straight or slightly flexuous, cylindrical, unbranched, smooth-walled, dark brown or black at the base, becoming paler to pale brown or subhyaline towards the apex, 103–364 µm long, (–3.5)5.8–10 µm wide at the base (x̄ = 206 × 8.2 μm, n = 20). ***Conidiogenous cells*** integrated, determinate, phialidic, with lateral phialides occasionally, cylindrical to lageniform, with indistinctive collarettes, smooth-walled, brown at the base and becoming subhyaline to hyaline towards the apex, 30–67.5 µm long × (3.2–)4.6–7 µm wide (x̄ = 43 × 5.8 μm, n = 20). ***Conidia*** amerospores, aseptate, reniform, often unilateral ventricose, guttulate, hyaline to subhyaline, 10–13 µm long × 4.5–7 µm wide (x̄ = 11 × 5.8 μm, n = 30), with unbranched, tubular, filiform, hyaline, 8.3–13.3 µm long setula at each end. **Sexual morph**: not observed.

#### Culture characteristics.

Conidia germinated on WA within 24 hours at 25 °C. Germ tubes were produced from the end. Colonies on PDA medium reaching ca. 28 mm diameter after one month, slightly irregularly circular, with nearly entire margin, flat, with a taupe protuberance in the center, off-white, with a taupe ring near to margin.

#### Material examined.

China • Guangxi Province, Yulin City, Minle Town, on dead rachis of an unidentified fern, 16 July 2023, S. Chen, 65 (GZAAS 23-0850, **holotype)**, ex-type GZCC 23-0769.

#### Additional sequence.

SSU: PX971511.

#### Notes.

Morphologically, *Codinaea yulinensis* fits well with the generic concept and is most similar to *C. dwaya* in lacking setae, possessing unbranched conidiophores and monophialidic conidiogenous cells. However, *Codinaea yulinensis* differs in producing reniform and often unilateral ventricose conidia with unbranched, hyaline setulae at each end, whereas *C. dwaya* has spherical to broadly oblong or pyriform conidia with setulae distributed over the surface (Réblová et al. 2021b; Zhang et al. 2022). Phylogenetically, *Codinaea yulinensis* forms a distinct clade, which shares sister relationship with the clade comprising *C. dwaya*, *C. pandanicola*, *C. siamensis*, and *C. trisetula* (Fig. 1). Comparisons of the ITS, LSU, and *tef*1-α gene regions further demonstrates sequence difference among these taxa (Table 2), supporting their recognition as distinct species (Jeewon and Hyde 2016).

**Table 2.** Comparison of nucleotide differences between *Codinaea yulinensis*GZCC 23-0769 and *C. dwaya* CBS 261.77, *C. pandanicola* KUMCC 16-0153, *C. siamensis*MFLUCC 15-0614 and *C. trisetula* NN044720.

Species	ITS	LSU	*tef*1-α
*C. dwaya dwaya* CBS 261.77	25/494 bp (5%, including 6 gaps)	10/826 bp (1.2%, including 1 gap)	44/909 bp (4.8%, including 5 gaps)
*C. pandanicola* KUMCC 16-0153	16/474 bp (3.3%, including 2 gaps)	5/804 bp (0.6%, including 1 gap)	26/850 bp (3%, without gaps)
*C. siamensis*MFLUCC 15-0614	22/503 bp (4.3%, including 4 gaps)	7/821 bp (0.8%, including 1 gap)	23/904 bp (2.5%, without gap)
*C. trisetula* NN044720	32/503 bp (6.3%, including 12 gaps)	9/829 bp (1%, including 5 gaps)	No available

### Pseudolomaantha yulinensis

Taxon classificationFungiChaetosphaerialesChaetosphaeriaceae

S. Chen, W. Zheng & L. Han sp. nov.

090405BC-0041-5763-9EE3-B5A4E88E704A

Index Fungorum: IF905054

Fig. 4

#### Holotype.

GZAAS 23-0851.

**Figure 4.**
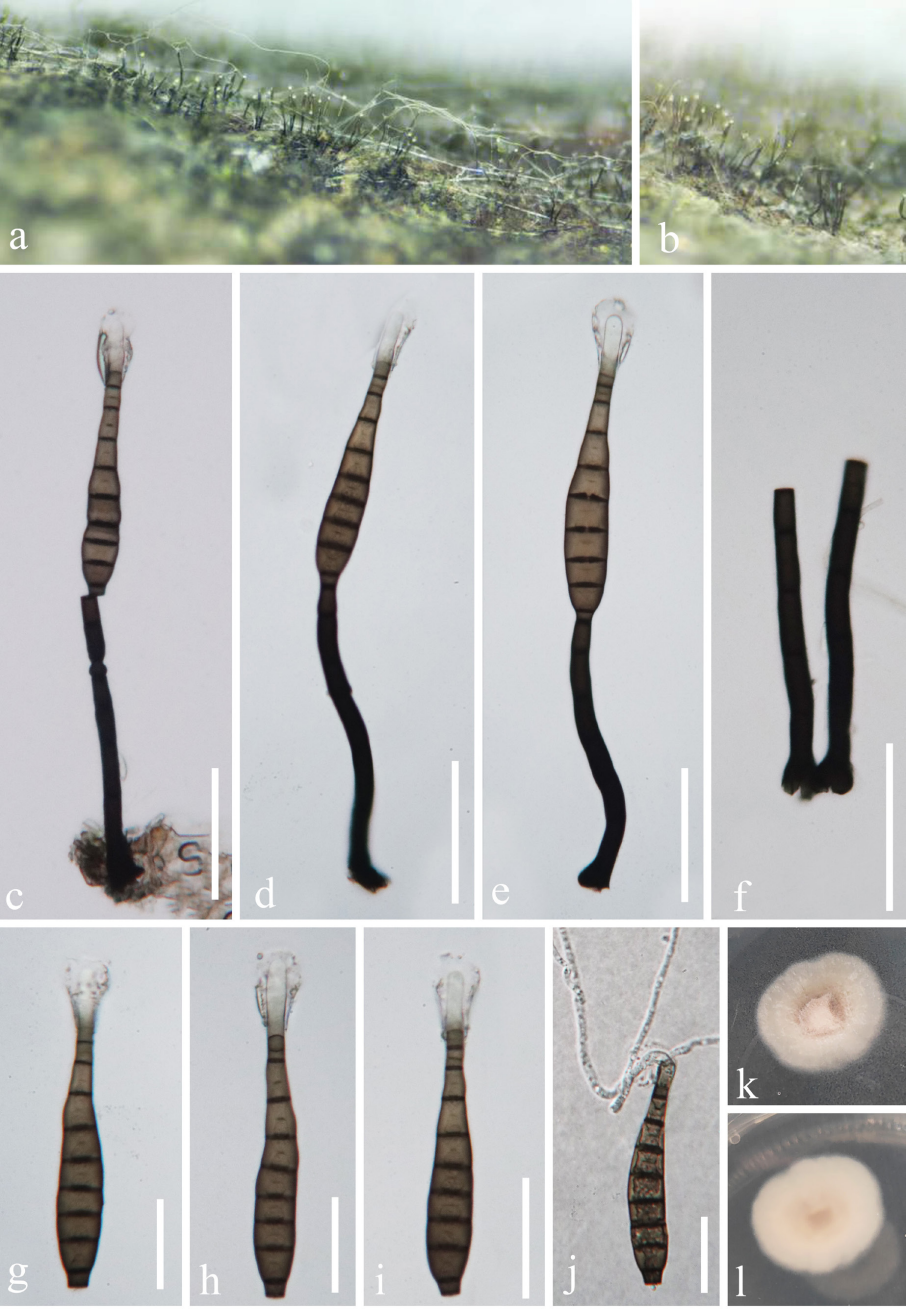
*Pseudolomaantha yulinensis* (GZAAS 23-0851, holotype). **a, b**. Colonies on the host substrate; **c–f**. Conidiophores with conidiogenous cells; **g–i**. Conidia; **j**. Germinating conidium; **k, l**. Pure culture from above and below. Scale bar: 50 µm (**c–f**); 30 µm (**g–j**).

#### Etymology.

The epithet “*yulinensis*” refers to the location (Yulin city) from which the fungus was collected.

#### Description.

***Saprobic*** on dead wood in a terrestrial habitat. ***Asexual morph Colonies*** on natural substrate superficial, effuse, scattered or gregarious, hairy, glistening, dark brown to black. Mycelium immersed mostly, composed of septate, smooth-walled, hyaline to brown hyphae. ***Conidiophores*** macronematous, mononematous, erect, solitary, septate, straight or slightly flexuous, cylindrical, dark brown or black, 92–110 × 6–9 µm (x̄ = 104 × 7 µm, n = 20). ***Conidiogenous cells*** integrated, terminal, holoblastic, monoblastic, sometimes elongating percurrently, cylindrical, dark brown or black, 7.6–13 × 6–7 µm (x̄ = 11.2 × 6.5 µm, n = 20). ***Conidia*** acrogenous, solitary, (9–)10(–11)-septate, with distoseptate, not constricted or slightly constricted at septum, straight or slightly curved, obclavate, rostrate, tapering towards the apex, truncate at base, guttulate, brown, subhyaline to hyaline at the apex, with gold and glistening appendages around the apex of the conidia, 100–115 × 13–17.5 µm (x̄ = 106.2 × 15 µm, n = 25). ***Conidial secession schizolytic*. Sexual morph**: not observed.

#### Culture characteristics.

Conidia germinating on WA medium within 24 h at 25 °C. Germ tube was produced from the apex of conidia. Colonies on PDA medium reaching ca. 22 mm in 20 days in dark, circular, edge entire, flat with a knobby protuberance, loose, flocculent, cream-colored.

#### Material examined.

China • Guangxi Province, Yulin City, Minle Town, on a dead wood in a forest nearby the roadside, 16 July 2023, S. Chen, 85.1 (GZAAS 23-0851, **holotype)**, ex-type GZCC 23-0770 = GZCC 23-0771.

#### Additional sequence.

SSU: PX971512 (GZCC 23-0770); SSU: PX971513 (GZCC 23-0771).

#### Notes.

*Pseudolomaantha yulinensis* forms a separate clade that is sister to *P. thailandica* with maximum support (100% ML and1 PP, Fig. 1). Morphologically, the former differs in producing shorter conidiophores (92–110 µm in *P. yulinensis* vs. 176–275 µm in *P. thailandica*) and smaller conidiogenous cells (7.6–13 in *P. yulinensis* vs. 12–22 µm in *P. thailandica*). In addition, there are 52 bp (10%, including 14 gaps, 511 bp) and 14 (1.7%, without gaps, 817 bp) differences between *P. yulinensis* (GZCC 23-0770) and *P. thailandica* (MFLUCC 24–0521) in the ITS and LSU regions. Therefore, we introduce *Pseudolomaantha yulinensis* as a novel species herein.

## Discussion

In this study, three new chaetosphaeriaceous species, *Brunneodinemasporium septatum*, *Codinaea yulinensis*, and *Pseudolomaantha yulinensis*, are introduced based on detailed morphological observations and multigene phylogenetic analyses. The first two species are characterized by phialidic asexual morphs, whereas the third possesses a non-phialidic, sporidesmium-like asexual morph. These findings provide further evidence of the high diversity of anamorphic chaetosphaeriaceous fungi in China (Zhang et al. 2022; Wu et al. 2022), a group that continues to yield new taxa despite increasing research attention. Notably, *Codinaea yulinensis* represents the first documented association of *Codinaea* with ferns (Zhang et al. 2025a), thereby extending the known host range of the genus.

In our phylogenetic analysis (Fig. 1), interspecific relationships within *Codinaea* lack strong statistical support, which is consistent with the recent studies (Réblová et al. 2021a, 2021b; Zhang et al. 2022; Kuo et al. 2024). This limited resolution may be attributed to the scarcity of molecular data for closely related taxa, many of which remain undiscovered or unsampled (Zhang et al. 2022; Hyde et al. 2024). In addition, although the present phylogenetic analyses incorporate both ribosomal (ITS and LSU) and protein-coding (*tef*1-α) markers, the absence of protein-coding sequences for many *Codinaea* species likely reduces phylogenetic signal and contributes to unresolved relationships. Future studies incorporating broader taxon sampling, re-examination of type materials, and multilocus phylogenetic analyses that include protein-coding loci will be essential for clarifying species boundaries and resolving evolutionary relationships within *Codinaea*.

Despite the limited number of accepted species, *Brunneodinemasporium* is currently known from relatively few taxa, and its reported distribution in Brazil and China may reflect limited sampling rather than true biogeographic restriction (Crous et al. 2012; Lu et al. 2016; Wu and Diao 2022). In contrast, *Pseudolomaantha* was previously known only from its type species in Thailand (Zhang et al. 2025b), and the present study constitutes the first report of the genus from China, markedly extending its known distribution. Expanded collections across regions, substrates, hosts, and habitats, combined with multigene phylogenetic analyses, are needed to better characterize the diversity, host range, and distribution of both genera.

## Supplementary Material

XML Treatment for Brunneodinemasporium septatum

XML Treatment for Codinaea yulinensis

XML Treatment for Pseudolomaantha yulinensis

## Additional information

### Use of AI

No use of AI was reported.

### Author ORCIDs

Sen Chen https://orcid.org/0000-0002-5872-2597

Haimei Zhao https://orcid.org/0009-0003-6300-0649

Wen Zheng https://orcid.org/0009-0002-8932-4564

Long Han https://orcid.org/0009-0002-7214-3652

### Data availability

All of the data that support the findings of this study are available in the main text.
